# Treating the Metabolic Syndrome by Fecal Transplantation—Current Status

**DOI:** 10.3390/biology10050447

**Published:** 2021-05-20

**Authors:** Stephen D. H. Malnick, David Fisher, Marina Somin, Manuela G. Neuman

**Affiliations:** 1 Department of Internal Medicine Cj Kaplan Medical Center, The Hebrew University, Rehovot 76100, Israel; stephen.malnick@clalit.org.il (S.D.H.M.); david.fisher@clalit.org.il (D.F.); marina.somin@clalit.org.il (M.S.); 2 In Vitro Drug Safety and Biotechnology, Banting Institute, University of Toronto, Toronto, ON M5G 0A3, Canada

**Keywords:** fecal transplantation, inflammatory mediators, metabolic syndrome, microbiome, metabolic-associated fatty liver disease, non-alcoholic fatty liver disease

## Abstract

**Simple Summary:**

The term “gut microbiome” refers to the microbial inhabitants whuch populate the intestine, including their number and diversity. In this review, we elaborate on how the microbiome affects the balance of pro- and anti-inflammatory responses in the gut. This ecosystem also influences systemic immunity. An imbalance of the microbiome is implicated in a number of inflammatory conditions, ranging from diabetes and metabolic disorders to non-alcoholic liver disease, cirrhosis, and liver cancer. A high dietary intake of animal-based processed foods and sugar is linked to a gut microbiome containing a higher volume of ‘opportunistic’ bacterial species, including the *Firmicutes* sp. and *Ruminococcus* sp., which are involved in pro-inflammatory activity. A diet rich in plant- and fish-based foods is linked to gut microbes that have the opposite effect by enhancing species such as the *Faecalibacterium* sp., which produce short-chain fatty acids that help control inflammation and protect the cells lining the gut. We also discuss fecal microbiome transplant as a modality to modify intestinal inflammatory processes via the changes in the gut microbiome.

**Abstract:**

The intestinal microbiome (IM) is important for normal gastrointestinal (GI) and other organ systems’ functioning. An alteration in the normal IM, dysbiosis, and changes in intestinal motility result in microorganisms’ overgrowth and an alteration in intestinal permeability. The gut–brain axis is also of importance in the irritable bowel syndrome (IBS) and associated bowel overgrowth. Secondary to the epidemic of obesity, the metabolic syndrome has become a major health problem. Disturbances in the fecal microbiome are associated with the metabolic syndrome. Metabolic-associated fatty liver disease (MAFLD) is now the current terminology for non-alcoholic fatty liver disease. IM alteration by fecal transplantation is an approved treatment method for recurrent *Clostridioides difficile* infection. Initially performed by either duodenal infusion or colonoscopy, it is now easily performed by the administration of capsules containing stools. We discuss the intestinal microbiome—its composition, as well as the qualitative changes of microbiome composition leading to inflammation. In addition, we discuss the evidence of the effect of fecal transplantation on the metabolic syndrome and MAFLD, as well as its clinical indications.

## 1. Introduction

In recent years, attention has been focused on the role of intestinal microbiomes inhabiting disease [1]. While there is no doubt that fecal microbial transplantation (FMT) is effective in treating *Clostridioides difficile* infection [2], its role in the treatment of other diseases is uncertain.

As a consequence of the epidemic of obesity, there is an increase in the prevalence of both the metabolic syndrome and MAFLD. The success of direct-acting antiviral treatment for chronic hepatitis C virus (HCV) infections has resulted in a decrease in the number of cases of HCV related cirrhosis and its complications. MAFLD is currently the major cause of cirrhosis in the USA; 30% of the population has MAFLD. We will discuss the evidence linking the microbiome to MAFLD, and the effect of FMT. We will also summarize the laboratory and clinical data implicating the microbiome in the pathogenesis of the metabolic syndrome and the current status of fecal transplantation as a potential treatment for the metabolic syndrome and MAFLD.

### 1.1. Gut Microbiota and Dysbiosis

The GI microbiome consists of vast numbers of organisms, including bacteria, fungi, archaea, and viruses [3]. A major portion of the microbiome is located in the ileum and proximal colon [4]. The multiple effects of the gut microbiota make it probable that disturbances will influence physiological function [4].

Gut microbial imbalance refers to alterations in the small intestinal bacteria, changes in the ratio of useful to harmful bacteria, and the translocation of colonic bacteria [5]. This is termed dysbiosis. As a result of interactions between microbes and their metabolites, the host immune response, physiological function, environment and diet in several disease states may be altered [5,6].

**Figure 1:**
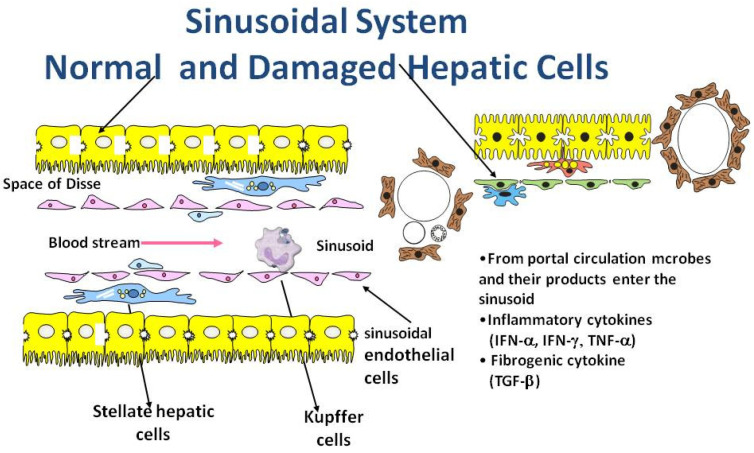
Hepatic sinusoid.

**Figure 2:**
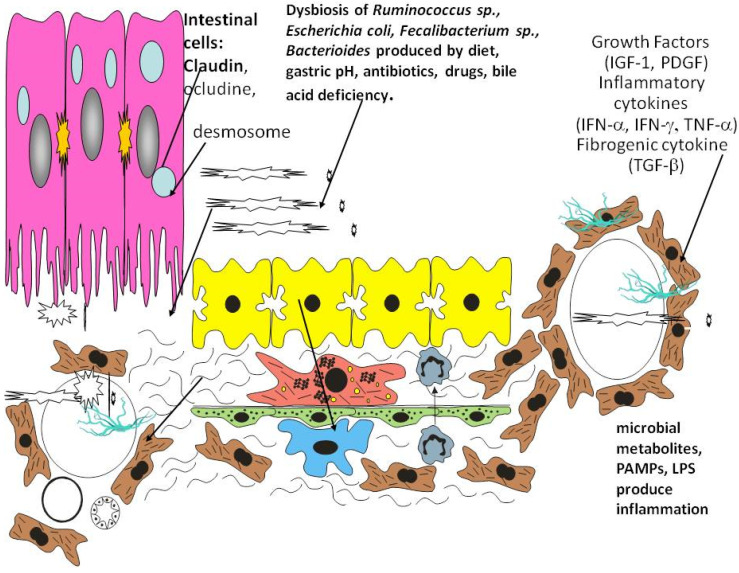
Intestinal microbiota influence the liver cell environment.

In Figure 1 and Figure 2 we illustrate how dysbiosis acts on the liver environment and alters the immune responses. The intestinal dysbiosis increases intestinal permeability to endotoxin and has a key role in the metabolic changes leading to pathogenesis. This results in the decreased production of long-chain fatty acids, which promote the growth of commensal Lactobacilli and maintain the integrity of the GI barrier. Hepatocytes, stellate cells, sinusoidal epithelial cells, and macrophages (Kupffer cells) are exposed to, microbes and their products including toxins and other metabolites via the circulation.

Healthy intestinal microbiota prevent pathogens from colonizing the intestine and contributes to immune responses. On the left side of Figure 1 can be seen normal liver tissue, normal hepatocytes, sinusoidal endothelial cells, hepatic stellate cells, and Kupffer cells. A cytokine release storm is the cellular immune response to toxic metabolites and changes in the microbiome.

On the right side of the figure, there are dysbiotic bacteria and viruses. The metabolic products and toxins produced by dysbiotic microbiome damage the hepatocytes and change the stellate cells in myofibroblasts. The hepatocytes lose some of the villi and stellate cells are transformed into myofibroblasts.

Changes in diet and lifestyle influence the microbiome. There is an increase of the Firmicute to Bacteroides ratio. An alteration to intestinal microbiome upregulates the alcohol-metabolizing enzyme cytochrome P450 2E1 and leads to endogenous alcohol production. Gut permeabilization permits pathogen-associated molecular patterns (PAMP) and endotoxin/lipopolysaccharide (LPS) entrance in portal circulation. An inflammation of the liver leads to the production of cytokines and chemokines.

In Figure 2 we present the intestinal cells surrounded by the microbiome that enters the hepatic circulation and produces changes in the liver cells and their micro-environment. Viruses and bacteria can be seen in the space of Disse and in the veins resulting in the production of proinflammatory cytokines and chemokines such as interferon alpha and gamma (IFN -α,γ), tumor necrosis factor alpha (TNF-α), as well as profibrinogenic cytokine transforming growth factor beta (TGFβ).

More than 50 different phyla are present in the gastrointestinal tract with different areas having different colonizations. Gram-positive organisms are predominant in the small intestine, whereas in the colon, the majority of bacteria are Gram-negative [4]. The bacterial population is regulated in part by the secretion of gastric and bile acid, by peristalsis, and the normal gut defense mechanisms [7]. In addition, extrinsic factors have an effect on the intestinal microbiome, including diet, bacterial and viral infections, motility-altering medications, pre- and probiotics, acid reducing medications, and antibiotics [8,9,10].

The importance of a healthy microbiome is underlined by its effect on preserving the epithelial barrier function [11,12,13,14]. Bacteria in the colon metabolize unabsorbed carbohydrates, resulting in the synthesis of short chain fatty acids (SCFA). The SCFA are an important energy source as a result of mucosal absorption [15]. This reduces the translocation of bacteria, the majority of which are potentially pathogenic, via the intestinal epithelial barrier [16].

### 1.2. Gut Microbiota and Intestinal Dysmotility

Small intestinal bacterial overgrowth impacts the intestinal microbiome. Both intrinsic and extrinsic factors regulate the content of the microbiome [17]. Factors regulating bacterial overgrowth involve intestinal tract secretions, including gastric and bile acid, peristalsis, mucin production, and gut antibacterial peptides (part of the normal gut defense mechanism). In addition, the ileocecal valve prevents bacterial retrograde translocation to the upper gut. Gastrointestinal motility is impacted by many factors, including diet, infections (both bacterial and viral), prokinetics, and medications which alter the IM such as proton pump inhibitors, histamine-2 blockers, and antibiotics [8,9,10].

Research on IBS has shown that changes in the microbiome contribute to motility disorders [18,19,20]. Patients with SIBO have different metabolomic profiles [21] as well as higher concentrations of lactate, acetate, and bile acids in comparison with control patients [22]. It has been suggested that small intestine bacteria overgrowth (SIBO) results in an excess production of both acetate and deconjugated bile acids. This causes a decrease in the intestinal epithelial cells ability to absorb SCFA. As a result, there is a decrease in the small intestinal barrier function [23].

### 1.3. Microbial Signaling, Gastrointestinal Motility, and Metabolic Disease

Microbial signaling refers to the translocation of bacterial metabolites or structural components from the epithelial cells of the intestine, which enables communication with other organ systems. PAMPs, including LPS, peptidoglycan, and flagellin, are detected by pattern recognition receptors. These include TLRs, retinoic acid-inducible gene-I-like receptors, and nucleotide-binding oligomerization domain-like receptors, found on both epithelial and immune cells and have a major role in host–microbe immune interactions [24]. There is also a crucial role for the aryl hydrocarbon receptor (AHR). The AHR is a transcription factor which influences responses to external stimuli. Both lactobacilli tryptophan ligand and indole-3 aldehyde are stimulated by the AHR [25]. In situations with dysbiosis, there are microbiota profiles which do not generate the AHR ligand, which results in metabolic disorders [26,27]. In addition, the AHR has been shown to function as a biosensor which affects intestinal motility via an interaction between the intestinal lumen on the programming of the enteric neural system [28]. The AHR regulates the microbe-associated intestinal peristalsis as part of the enteric nervous system surveillance pattern. Taken together, it appears that gut microbial dysbiosis produces changes in the host immune signals resulting in disorders of GI motility and metabolism. Modulating AHR signaling through changes in the gut microbiome may be a novel treatment for metabolic diseases.

Microbial metabolites are produced as a result of the degradation of dietary fiber, resulting in the production of SCFAs [29]

These SCFAs, including butyrate, propionate, and acetate, act as multi-functional signals binding to the G protein-coupled receptors (GPR43 and GPR41), also known as free fatty acid receptor 2 and 3 (FFAR2 and FFAR3). The binding of SCFAs to small intestinal and colonic FFAR2 and FFAR3 activates the secretion of L-cells GLP-1, which impacts insulin release and appetite [30,31]. SCFAs have a critical role in gluconeogenesis [32]. The importance of SCFA to colonic health is apparent from a report of the beneficial effect of oral SCFA administration to a hunger striker who developed starvation colitis [33]. In summary, SCFA production aided by the gut microbiome has an important role in GI motility and metabolic function.

## 2. Gastrointestinal Microbiome

### 2.1. Gut Microbiota and the Gut–Brain Axis

There is a reciprocal relationship between the brain and the gut [34]. A well-known example is the routine use of antibiotic therapy for hepatic encephalopathy [35]. The gut microbiota can influence brain function [36]. Furthermore, bi-directional gut–brain interactions have an important effect on several aspects of the functioning of the GI tract [37]. This interaction is an emerging area of research and the subject of a recent review [38].

A small proportion of patients with metabolic disorders have gastrointestinal dysmotility [39,40]. Microbial dysbiosis results in an impaired intestinal barrier function. Dysmotility may be the link to dysbiosis in these individuals. In addition, hyperglycemia has been shown to increase the permeability of the intestinal barrier through an alteration in tight junction integrity via the transcriptional reprogramming of the GLUT2-dependent epithelial cell [41]. Furthermore, the damage to the intestinal barrier causes islet-reactive T cell activation and autoimmune diabetes [42].

### 2.2. Metabolic Disorders and Gut Dysbiosis

Since gut microbiota influences metabolic homeostasis, it is to be expected that microbial dysbiosis may result in metabolic changes. These metabolic disturbances are mediated by changes in the gut barrier, resulting in metabolic inflammation [6,43,44]. In addition, the gut microbiota generates signaling molecules, which regulate energy production from indigestible carbohydrates [45,46].

Antibiotics can profoundly alter the microbial population, and this may result in metabolic disease [1]. This effect is more pronounced following exposure to antibiotics in early life. Antibiotic use in obese patients decreases peripheral insulin resistance [47]. Thus, there is a strong connection between the intestinal microbiome and central components of the metabolic syndrome. It is therefore of great interest to examine the effect of the alteration of the microbiome on the development and progression of the metabolic syndrome and its effect on the liver.

### 2.3. MAFLD, the Metabolic Syndrome, and the Relation to the Intestinal Microbiome

**Figure 3:**
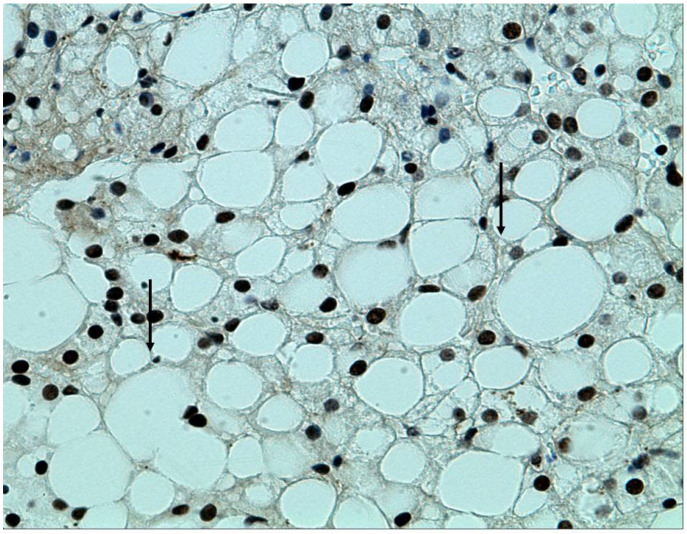
Immunohistochemistry (stain—caspase-cleaved K18 (ccK18); Bender MedSystems (Vienna, Austria)). Cytokeratin 18 (arrows) stains the cell death by apoptosis. The hepatocytes present large lipid droplets that occupy 80–90% of the parenchymal cells.

The metabolic syndrome has several different definitions, but they all include obesity, increased abdominal circumference, hypertension, insulin resistance, and a disturbed lipid profile [48]. MAFLD progresses from steatosis to steatohepatitis and fibrosis, resulting in cirrhosis and its complications, including hepatocellular carcinoma. In Figure 3 we present a liver biopsy of a patient with steatohepatitis.

Liver biopsy has been considered the “gold standard” in the study of liver disease. In clinical practice, in the absence of decompensated liver disease, liver biopsy is suitable for establishing the diagnosis of NAFLD/NASH, assessing the stage and prognosis, and excluding concomitant and/or other causes of damage.

Liver biopsy is an expensive invasive procedure with potential complications, and it is not recommended for all patients with suspected NAFLD. However, it is useful for establishing the stage and severity of MALD/NAFLD, in case of aggressive forms or severe steatohepatitis requiring specific therapies, and for distinguishing other causes of liver disease. The typical histological features in patients with NAFLD include steatosis, hepatocellular ballooning, lobular polymorphonuclear cell infiltration and inflammation, fibrosis, lobular distortion, and cirrhosis.

The IM influences the development of MAFLD. Initial studies centered on the examination of the fecal microbiome in patients with MAFLD and its comparison to that in healthy individuals. Classic microbiological techniques based on culture are not sufficiently sensitive to characterize the fecal microbiome, and the recent use of high throughput sequencing technology (shotgun sequencing or pyrosequencing) has made substantial contributions to this field. In addition, decreasing costs and analytical turnover time have made available large amounts of data, which enable efficient microbiome studies with the use of advanced bioinformatic techniques. The transfer of human gut microbiome from an obese twin to germ-free mice resulted in a metabolic phenotype characteristic of obesity. This effect was ameliorated when the mice were co-housed with mice exposed to the microbiome of the lean twin [49]. This underlines an intimate connection between the development of obesity and the human microbiome. Furthermore, germ-free mice have less weight gain than normal mice when given a high sugar and fat diet, even when consuming a high amount of food [50].

In addition, germ-free mice receiving a high-fat diet are more sensitive to insulin, and germ-free mice colonized with the IM from conventional mice have an increased body fat content. Further evidence suggesting the important role of the microbiome in obesity and insulin resistance include transferring the insulin resistance index by FMT [51], which has been shown to affect the accumulation of fat in macrophages and glucose metabolism, but by distinct mechanisms [51]. Moreover, in a mouse model of diet-induced obesity, the administration of antibiotics improved fasting glycemia and insulin resistance independently of food consumption or degree of adiposity [52]. Both hepatic lipogenesis and steatosis were diminished following antibiotic administration [52].

The transplantation of IM from lean male donors to males with the metabolic syndrome resulted in a decrease in insulin resistance [53]. Furthermore, the IM from obese individuals has been shown to have a different microbial signature and diversity from that of lean people. For example, they have less *Bacteroides* and more *Firmicutes* [54].

Specific microbiome signatures characteristic of both obesity and type 2 diabetes mellitus and their complications have been described, suggesting that an IM dysbiosis is present in metabolic diseases. This has recently been the subject of an extensive review published elsewhere [55]. There is also a specific microbiome—based on the metagenomic signature associated with advanced fibrosis in patients with NAFLD—that raises the possibility of microbiome studies as a potential replacement for determining cirrhosis [56].

## 3. Role of Inflammation

Changes in microbiome are seen in both DAFLD and MAFLD [57]. The gut microbiome is a contributing factor in MAFLD and ALD [58,59,60]. An increase in the absorption of gut endotoxins and their translocation via the portal vein to the liver is well recognized in ALD. Alcohol intake may alter gut bacteria [61,62]. The resulting intestinal dysbiosis together with increased intestinal permeability are important in the development of ALD, MAFLD, and the severity of cirrhosis [63]. Alcohol increases the gut permeability to LPS. Alcoholic liver injury is a consequence of the toxic effects of reactive oxygen species produced by ethanol-induced cytochrome P450 2E1, as well as acetaldehyde-altered proteins [64]. It is associated with a decrease in the production of long-chain fatty acids, which support both the growth of commensal Lactobacilli and gut barrier integrity. The altered IM and the subsequent intestinal damage result in an increase in endotoxin uptake. These endotoxins may then link to TLR4 and CD10 receptors present in Kupffer cells and activate nuclear factor κB (NF-κB), resulting in the release of proinflammatory cytokines (II-8, II-6, TNFα, II-1ß) and chemokines (CC-chemokine ligand 2 (CCL2)), which induce inflammation [65]. Administration of antibiotics, together with Kupffer-cell destruction, may improve the dysbiosis. We have previously shown that the translocation of gut bacteria across the epithelium increases dysbiosis [65]. A number of translocated commensal bacteria in a healthy human gut are neutralized by Th1 and Th17 cells, which are induced by mucosa-adherent bacteria and the polysaccharides of *Bacteroides* sp. Invading bacteria continuously activate TLRs, resulting in an overexpression of pro-inflammatory cytokines, in which subsequently damages the gut epithelium and causes chronic inflammation [66]. Both MAFLD and autoimmune diabetes are associated with chronic inflammation. An intestinal dysbiosis affects the maturation of the innate immune system. The function of both neutrophils and dendritic cells is reduced in the absence of an intact immune system, thus resulting in a decrease in the number of pathogens that are killed. This is coupled with a reduced secretion of type I interferons (IFN-I) and interleukin (IL-15). As a result, the clearance of systemic infections is decreased. Gut homeostasis is dependent on the immune system of both the host and the IM. An imbalance in this interaction results in an increased risk of immune-related diseases [66,67]

## 4. FMT as Treatment for the Metabolic Syndrome

FMT was first reported during the Dong Jin dynasty in China, nearly 1700 years ago [68]. FMT was used in modern medicine in the 1950s at Johns Hopkins in Baltimore, where fecal enemas were administered to patients with pseudomembranous colitis [69]. More recently, Larry Brandt in New York has promoted FMT for patients with *Clostridioides difficile* infection, with the use of stools donated by the patient’s partner [70]. FMT is the treatment of choice for recurrent *Clostridioides difficile* infection [71].

FMT has also been investigated for other diseases, including inflammatory bowel disease (IBD), irritable bowel syndrome (IBS), kidney disease, the metabolic syndrome and some of its components, and MAFLD [72,73,74]. Dysbiosis of the IM describes an alteration in the gut bacteria as compared to those in healthy individuals. This results in an unbalanced microbial population, with lessened diversity and a decrease in metabolites, including SCFA.

A systematic review of FMT as treatment for both obesity and the MS (up to December 2018) found three randomized double-blind placebo-controlled published studies, which included 76 subjects with obesity and the MS [75]. These studies all involved male patients. One had 9 patients [53], another had 26 patients [43], and the last involved 10 patients [76]. The mean BMI was 34.8 ± 4.1 kg/m^2^. Two studies found an increase in peripheral insulin sensitivity at 6 weeks following donor FMT as compared to that in patients receiving placebo. One study reported a decrease in hemoglobin A1c (HbA1c) levels in patients who received FMT after six weeks.

However, fasting plasma glucose levels, hepatic insulin sensitivity, body mass index, and serum lipids were similar in the two groups in all these studies. The single study that reported an increase in the rate of glucose disappearance and HbA1c did not find that this was maintained over the 18-week study period of the trial [43]. Furthermore, other important markers, including BMI, fasting plasma glucose, triglycerides, and HDL and LDL cholesterol, showed no improvement between the two groups.

In summary, these studies showed a transient improvement in insulin sensitivity without an improvement in other clinical parameters. The FMT showed no improvement in the microbial alpha diversity Shannon index in the obese patients.

One of the problems with these early studies was that the route of administration of the transplanted fecal material was by invasive endoscopic procedures [77]. More recently, fecal administration by capsules has become available. Stool is obtained either as fresh fecal material or as lyophilized stool, which are encapsulated to prevent digestion by gastric acid [78]. It is also more aesthetically pleasing and acceptable to those patients who receive it [78,79]. This has proven effective for the treatment of recurrent *Clostridioides difficile* infection and has the advantage that it is not invasive and can easily be repeated.

A recent double-blind study has been published, which involved 22 patients with a BMI greater than 35 kg/m^2^, without diabetes, NASH, or MS, and treated by FMT in the form of capsules from a lean donor [80]. The patients received 30 capsules at week 4, and another 12 capsules at week 8, and were compared to a placebo capsule group during a follow-up period of 26 weeks. The primary outcome of this study was safety, although both stool and serum were tested by 16S ribonucleic acid (RNA) sequencing and metabolomes by liquid chromatography—mass spectrometry. In addition, changes in the area under the curve for GLP-1 after 12 weeks were examined. The administration of the capsules was safe, although it did not result in a decrease of BMI in the patients who were obese but metabolically normal. There were prolonged changes in the IM bile acid profiles, resembling that of the single lean.

There were no changes in GLP-1, but there was a change in nine operational taxonomic units (OTUs) in the genus *Faecil bacterium,* which were both butyrate-producing and bile-hydrolyzing. This may result in a decrease in primary bile acids. Bile acids increase fat absorption and have a role in glucose lipid and energy balance. This is important in the development of obesity. Theoretically, an increase in butyrate-producing organisms following FMT might be expected; however, this was not seen in either stool or serum. This was also not found in the previous metabolic syndrome studies [81].

We also carried out an unpublished study of FMT in overweight and obese patients undergoing screening colonoscopy. Fecal material obtained from a single lean donor was injected into the colon (and the terminal ileum) upon withdrawal during a screening colonoscopy. A total of 20 patients were included in the study, who were then compared to 20 non-obese patients who underwent screening colonoscopy with saline injection on withdrawal. There was a high dropout rate and only 10 patients were available for analysis. Although there was no prolonged weight loss, there was a significant decrease in the abdominal circumference.

There are other reports examining the use of fecal capsules in obesity and its effect on the IM. The FMT-TRIM trial was a placebo-controlled trial from a single academic medical center in the United States [80]. There were 24 obese adults with mild to moderate insulin resistance who were randomized to receive either FMT from a healthy lean donor or placebo capsules for 6 weeks. No significant improvements in insulin sensitivity, homeostatic model assessment-insulin resistance (HOMA-IR), or body composition were found. The donor bacterial groups achieved a variable degree of engraftment, and there were no serious adverse events during the 12-week follow up [82].

Attempts have been made to use bacterial probiotics in subjects with either obesity, diabetes, or MAFLD. A systematic review and meta-analysis in 2019 involving 105 articles found small but maintained improvements in several metabolic risk factors. In overweight but not obese participants, there were significant decreases in several parameters, including weight, BMI, waist circumference, and visceral adiposity. In type 2 diabetics, the probiotics resulted in a decreased fasting glucose, a decrease in glycated hemoglobin levels, and a decrease in HOMA-IR. The patients with MAFLD had a decrease in ALT and AST levels [83,84]. There are additional reports involving the administration of differing bacterial species that have improved some metabolic determinants in obese patients [85].

Trimethyl-amine-N-oxide (TMAO) is a metabolite derived from the bacterial population of the intestine that is involved in atherosclerosis [86]. Plasma levels of TMAO are related to cardiovascular disease in animal models [87].

Individuals who consume a vegan diet have a decreased capacity to produce TMAO, which may be linked to a modified IM [76]. FMT’s effect on TMAO has been examined in a pilot study of 20 males with MS who were randomized to receive fecal transplantation from either a single lean vegan donor or autologous transplantation. The ability to produce TMAO was determined at baseline, and after 14 days by a choline and carnitine challenge test, using isotope-labeled d_6_-choline and d_3_-carnitine. Fresh stools donated by both the single vegan donor and the patients were gathered on the day of the treatment and administered by nasoduodenal infusion. There was a significant difference in the intestinal microbiota composition between the patients with the metabolic syndrome and the vegan patients. Following transplantation, the fecal microbial profile in the metabolic syndrome patients transplanted with stool from a lean vegan showed a transformation to the vegan profile in some cases, although there was no change in fecal microbiota diversity. However, there were no functional effects on TMAO production [88]. This has a size limitation in both the patient population and the length of follow-up.

Bariatric surgery is now an accepted treatment for morbid obesity and has been shown to decrease mortality [89]. In a study on diet-induced obesity (DIO) in mice, FMT from post-bariatric surgery DIO donors to DIO recipients resulted in significant weight loss [90]. In addition, patients who received an FMT from patients who have undergone a Roux-en-Y gastric bypass or vertical banded gastroplasty had an improved metabolic state [91].

Diabetes mellitus (DM) is closely related to the metabolic syndrome, and type 1 DM is associated with changes in the IM [91]. A recent study has found that FMT halted the progression of new-onset type 1 DM. A total of 17 patients aged 18 to 30, with an onset of less than six weeks of type 1 DM, received three autologous FMTs over a 3-month period. They were compared to a group receiving three FMTs from same-gender healthy donors. A follow up after 12 months showed that the stimulated C peptide levels were more preserved in the group that received autologous FMT as compared to the group that received healthy donor FMT. This study demonstrates in patients recently diagnosed with type 1 DM that FMT delays the decrease in production of endogenous insulin. There was also an inverse relation between small intestinal *Prevotella* (as assessed from duodenal biopsy at endoscopy) and residual beta cell function [92].

A recent study from Israel examining FMT in patients with weight regain together with the recurrence of cardio-metabolic factors after rapid weight loss has been published [93]. Ninety patients working in the same workplace participated in a weight loss trial. Inclusion criteria included abdominal obesity or dyslipidemia. They were divided into three groups: group 1, appropriate dietary guidelines; group 2, the Mediterranean diet; and group 3, a green Mediterranean diet. The participants on the green Mediterranean diet received green tea and a supplement of *Wolffia globosa* (Mankai strain). Upon completion of the 6-month diet, a mean weight loss of 8.3 kg was achieved. Fecal samples were obtained and processed into capsules at this time. The study subjects then received capsules with either their own stool or placebo up to month 14. The FMT green Mediterranean group had a significantly smaller weight regain (17.1% vs. 50% placebo, *p* = 0.02). In addition, there was a smaller gain in the waist circumference and insulin rebound in the green tea group. Furthermore, only the green tea group had a significant change in the IM bacterial population.

FMT is employed to restore the microbiome. Patients who underwent hematopoietic stem cell transplantation together with autologous FMT after antibiotic therapy (with stool stored before starting antibiotic therapy) were shown to have a restoration of both microbial diversity and composition [94].

The field of the intestinal microbiome is rapidly developing, but it is certainly not yet prime time for therapy. The evidence is incomplete. It is probably not a one-size-fits-all situation. It is possible that the recipient may need to have his baseline microbiota assessed in order to determine which strains promote microbiomal recovery and which may be lacking in the specific patient due to have the FMT [95]. It should also be remembered that the feces that is transplanted additionally contains fungi, archaea, bacteriophages, and metabolites, and there is a lack of data regarding their function [96].

## 5. Conclusions

The IM plays an important role in maintaining the integrity of the intestinal epithelial barrier and extracting energy from ingested food. Alterations in the IM have been detected in obesity and the metabolic syndrome, and have been shown to have a role in the development of the MS.

In addition, the IM has an important modulating effect on immunologic factors that regulate intestinal homeostasis and mucosal inflammation. Cytokines, which regulate of leukocyte translocation and apoptotic cell death, are now recognized as essential immune molecules in the pathogenesis of the MS and other diseases.

Manipulation of the FM by adapting a healthy lifestyle, including dietary changes and exercise, have been linked to an improvement in hepatic damage in MAFLD. Other factors influence the IM, including probiotics and antibiotics. FM is an approved therapy for the treatment of relapsing *Clostridioides difficile*. Clinical studies are being performed on FMT for the treatment of several disease states, including MAFLD, obesity, irritable bowel syndrome, and inflammatory bowel disease.

A better understanding of the dynamic interaction between cytokines and microbiota diversity, together with the implications for both immunity and inflammation, is necessary in order to examine the potential use of the manipulation of the IM by FMT in the treatment of MS and other disorders.

## Data Availability

The data presented in this study are openly available in each one of the cited articles.

## Abbreviations

ALD	Alcoholic liver disease
AHR	Aryl hydrogen receptor
BMI	Body mass index
DAFLD	Dysbiosis-associated fatty liver disease;
DIO	Diet-induced obesity
FFAR 2 and 3	Free fatty acid receptor 2 and 3
FMT	Fecal microbial transplantation
FMT- TRIM	Fecal microbiota transplantation for the improvement of metabolism in obesity
GI	Gastrointestinal tract
GLP-1	Glucagon-like peptide-1 = gluco-regulatory incretin like hormone
GLUT-2	Glucose transporter 2
GPR 41 and 43	G protein coupled receptor 41and 43
HbAc1	Hemoglobin Ac1
HDL	High density lipoprotein
HOMA-IR	Homeostatic model assessment-insulin resistance
IBD	Inflammatory bowel disease
IBS	Irritable bowel syndrome
IFNα,γ	Interferon alpha, gamma
IM	Intestinal microbiome
L cells	Enteroendocrine L cells.
LDL	Low density lipoprotein
LPS	Lipopolysaccharide
MAFLD	Metabolic associated fatty liver disease
MS	Metabolic syndrome
NAFLD	Nonalcoholic fatty liver disease
NFκB	Nuclear factor kappa B
OTUs	Operational taxonomic units
PAMP	Pathogen associated molecular pattern
SCFA	Short chain fatty acids
SIBO	Small intestine bacterial overgrowth
TLR	Toll-like receptor
TMAO	Trimethyl-amine-N-oxide
TGFβ	Transforming growth factor beta
TNFα	Tumor necrosis factor alpha
